# A Historical Sketch and Some Aspects of Sterility

**Published:** 1920-12-25

**Authors:** 


### THE VOGUE OF ORGANO-THERAPY.

### A Historical Sketch and Some Aspects of Sterility.

The earliest notes of a belief in the curative power of animal tissues, or of extracts made from them, may be traced almost as far back as ancient literature will take us. The Persians, Egyptians, Greeks, and Romans all recorded and advocated their use in the treatment of disease. Andromachus, imperial physician to Nero, together with Pliny, Hippocrates, and Galen, all mentioned this form of therapy in considerable, if somewhat anomalous, detail, the main bearing of which was that disordered vital organs might be corrected by the ingestion of normal organs, and where the disorder was complex in its origin then what we now call a pluriglandular product must be given. The sixteenth-century medical writings are rich in prescriptions containing such items as wolf liver, inner membrane of chicken stomach, etc., and the apothecary of that time was called upon to provide, if necessary, even "Cranium Humanum!"

There was at that time an essential difference between the ancients' theories and modern ideas on endocrine secretions, for the reputed efficacy was then based simply on the ancient dogma attributing to all animal tissues automatic functional potentialities, the substance of a given organ exercising a specific stimulating effect upon the function of a similar organ in the patient. Thus the "Elixir of Youth" was administered to both sexes for obvious purposes, and in view of sundry recent so-called discoveries, there is little doubt that actual therapeutic effects were obtained.

But many quaint, sometimes terribly erroneous, customs of the past may be directly ascribed to this early idea of specific organo-therapy, such as the selective cannibalism at one time practised by certain Oriental tribes, with the object of acquiring the endowments attributed to certain human organs; and there is a span of many hundred years before such chaotic ideas gave place to the work of Brown-Séquard, who may be said to have initiated the first impetus given to endocrinology as considered in the medical world to-day.

### The Modern Tendency.

Having given, therefore, the briefest indications of how old in truth is our latest medical fashion, it might be well to define somewhat more clearly the present position, and in particular the degree of justification with which popular faith may be fixed upon these glandular extracts in general. Here these notes are indebted to Dr. Sturmdorf's writings in the well-known American publication *International Clinics* (Lippincott) and to those more deeply interested in the subject consideration of his writing is well worth while. But for the moment, to give some defined field for discussion, let the question as it affects female sterility suffice.

Obviously, in the first place, to know when endocrines are indicated the practising profession must know why and how they act. It is not an unwarrantable criticism to say that this knowledge is not widely possessed.

Also it is remarkable how frequently one hears of gland extract administration being attempted without a preliminary review of the simple physiological abnormality or otherwise of the patient concerned. From a therapeutic point of view, the term "sterility" is purely relative, necessarily implying in a given case the presence of approximately normal anatomic and physiological essentials to conception, but without the consummation of offspring. It is needless to recount the details of these essentials, but it must be strongly urged that an endocrine substance cannot be expected to *create* a function; it can only tend to activate one existing in a latent state, stimulate one that is deficient, or possibly mobilise one that is temporarily inhibited.

Again, leaving aside the grosser physiological defects, there are many less understood factors which cannot necessarily be expected to yield to any sudden influx of an endocrine substance. To quote from the literature already mentioned, the maturation of a Graafian follicle and liberation of its contained ovum, the evolution of the corpus luteum, and the subtle factors that dominate ovular fertilisation, are all susceptible to inhibiting influences, temporary or permanent, local or systemic, most of which involve problems far beyond our diagnostic horizon. Who can explain why the conjugation of a perfectly normal female, with an equally normal male proves sterile, while subsequent union of each with another demonstrates the fertility of both. To apply the serological hypothesis of "a selective ovular immunity to certain strains of spermatoza" in explanation, is mere terminological juggling.

In brief, then, it is simply the development and degree of functional activity of the reproductive gland which can be influenced by the endocrine substances. Just as sex is apparently predetermined in the ovum, it appears more than probable that the fertility or sterility of the cell is also predetermined and quite independent of direct endocrine influence; and to make this point clearer, one might well sketch the whole glandular history of the human being, in infancy, youth, puberty, pregnancy, etc. In each case the limitations, as well as the remarkable powers of the various endocrine substances, are noticeable.

Therefore, without labouring these details, it becomes more obvious that there are surprisingly numerous difficulties surrounding the accurate diagnosis of what may be termed endocrine sterility. Such a diagnosis obviously can be made only after the exclusion of every other pathogenic factor or physiological abnormality and the discovery of evident and pathognomonic stigmata of endocrine deficiency.

**The Vogue of Organo-Therapy**—(*continued*).

Diagnosis in Practice.

The watch, then, is to be for a symptom complex, and in the typical instance there is no need to pretend the discovery is difficult. No one would attempt to treat as obscure the sterility of advanced acromegaly, Graves' disease, myxœdema, or Addison's disease. The results are, however, here usually irremediable from the reproductive point of view. It is only in the ill-defined, obscure, but suggestive instance of endocrine disorder that this phase of organo-therapy comes truly into its own.

At the moment, there is one outstanding fact before the profession—and progress beyond it is, as yet, slight. In the human body under normal conditions exists what is more or less aptly called the "endocrine chain." In this chain, at any given period of human existence, the various ductless glands must exactly co-ordinate their activities. Each must, at any moment, be performing what is required of it, no more and no less. If this chain is perfect, then other conditions, being normal, sterility is not due to endocrine disorder. But if for any reason the running of the chain is broken, or permanently or progressively becomes defective, then one of the observable results *may* be sterility.

The question, therefore, naturally arises as to how such a break may occur, and in summary, the present state of knowledge provides two explanations. The first occurs when one member of the gland series is destroyed or invaded by some absolutely extraneous pathogenic progress, the other when intrinsic disease occurs in a gland itself, and automatically the endocrine balance is broken by deficiency or over secretion on the part of one of its members. Exactly how and whether any particularly pathological happening will produce sterility is a question that the years to come must answer. In certain well-defined instances it is possible to replace a deficient secretion by organo-therapy and many excellent examples of success might be given. But on the other hand, to treat every case of sterility with endocrines regardless of its pathogenesis, which, if not openly advocated, is not infrequently done, is about as rational as to dilate, curette, or incise every cervix for the same purpose, and would yield probably about the same percentage of good—and bad—results. At the moment it should be realised that "endo-crinopathic" sterility is not necessarily connected with a primary ovarian deficiency. Also whether it is due to the absence or the presence of an inhibiting hermone, truthfully, we *do not know*, and cannot therefore formulate exact therapeutic indications.

Again, to quote Dr. Sturmdorf, "Many women undoubtedly conceive during a course of organo-therapeutic experimentation, as they do after other methods of treatment, rational and irrational, but to attribute an eventual fecundity to the curative effect of this or that procedure, simply because it so happened, is an obvious *post hoc ergo propter hoc* interpretation without substantiation, for it must be conceded that many women present identical indications with or without endocrine or other treatment, while on the other hand, a very large proportion of these so treated remains sterile. When all is said that can be said to-day of the endocrines in sterility, the entire subject may be epitomised as a treatment of a condition of which we know little, by a means of which we know less."

